# Hospital Readmissions of Patients with Heart Failure: The Impact of Hospital and Primary Care Organizational Factors in Northern Italy

**DOI:** 10.1371/journal.pone.0127796

**Published:** 2015-05-26

**Authors:** Vera Maria Avaldi, Jacopo Lenzi, Ilaria Castaldini, Stefano Urbinati, Giuseppe Di Pasquale, Mara Morini, Adalgisa Protonotari, Aldo Pietro Maggioni, Maria Pia Fantini

**Affiliations:** 1 Department of Biomedical and Neuromotor Sciences, Alma Mater Studiorum—University of Bologna, Bologna, Italy; 2 Department of Programming and Control, Bologna Local Healthcare Authority, Bologna, Italy; 3 Department of Cardiology, Bellaria Hospital, Bologna, Italy; 4 Department of Cardiology, Maggiore Hospital, Bologna, Italy; 5 Department of Primary Care, Bologna Local Healthcare Authority, Bologna, Italy; 6 ANMCO Research Center, Florence, Italy; University of Chieti, ITALY

## Abstract

**Background:**

Primary health care is essential for an appropriate management of heart failure (HF), a disease which is a major clinical and public health issue and a leading cause of hospitalization. The aim of this study was to evaluate the impact of different organizational factors on readmissions of patients with HF.

**Methods:**

The study population included elderly resident in the Local Health Authority of Bologna (Northern Italy) and discharged with a diagnosis of HF from January to December 2010. Unplanned hospital readmissions were measured in four timeframes: 30 (short-term), 90 (medium-term), 180 (mid-long-term), and 365 days (long-term). Using multivariable multilevel Poisson regression analyses, we investigated the association between readmissions and organizational factors (discharge from a cardiology department, general practitioners’ monodisciplinary organizational arrangement, and implementation of a specific HF care pathway).

**Results:**

The 1873 study patients had a median age of 83 years (interquartile range 77–87) and 55.5% were females; 52.0% were readmitted to the hospital for any reason after a year, while 20.1% were readmitted for HF. The presence of a HF care pathway was the only factor significantly associated with a lower risk of readmission for HF in the short-, medium-, mid-long- and long-term period (short-term: IRR [incidence rate ratio]=0.57, 95%CI [confidence interval]=0.35–0.92; medium-term: IRR=0.70, 95%CI=0.51–0.96; mid-long-term: IRR=0.79, 95%CI=0.64–0.98; long-term: IRR=0.82, 95%CI=0.67–0.99), and with a lower risk of all-cause readmission in the short-term period (IRR=0.73, 95%CI=0.57–0.94).

**Conclusion:**

Our study shows that the HF care specific pathway implemented at the primary care level was associated with lower readmission rate for HF in each timeframe, and also with lower readmission rate for all causes in the short-term period. Our results suggest that the engagement of primary care professionals starting from the early post-discharge period may be relevant in the management of patients with HF.

## Introduction

Heart failure (HF) is a major clinical and public health issue and a leading cause of hospitalization, with a prevalence ranging from 1–2% in the general adult population of developed countries and increasing to 10–20% in people older than 75 years of age [1]. HF is characterized by high mortality and morbidity, and poor quality of life. Patients with HF are frequently readmitted to the hospital not only because of progression of the underlying disease, but also for complications due to poor adherence to pharmacological treatment, worsening of comorbidities, poor self-care, and inadequate support. Direct and indirect costs of HF are mostly due to hospitalizations [2].

Many studies have suggested that primary health care should be the core of good management of chronic diseases, such as HF, and underlined the value of multidisciplinary interventions to cope with the complex needs of patients with chronic conditions [3–7].

In the past few decades, several strategies have been implemented for HF management. Randomized controlled trials and several observational studies have shown that a multidisciplinary care approach [8–10], case management interventions [11–15], and telemonitoring programmes [16] may reduce the incidence of readmission, death, and eventually related costs [17]. The purpose of these programmes is to promote patient education to the early detection of signs of worsening HF, adherence to evidence-based pharmacological treatment, and the interplay between general practitioners, specialists, nurses, and other care professionals including caregivers [18,19]. However, given the high heterogeneity between the components of the interventions, their duration and the patient populations [20], the results of these studies are frequently contradictory, making it difficult to ascertain whether programmes work or not. Thus, it is necessary to identify the key elements in the management of patients with HF that are more effective and efficient in each specific context [21,22].

In Italy, where the National Health Care System offers a universal coverage, primary care is provided by Local Health Authorities (LHAs), a network of population-based health management organizations including the hospitals and community services of specific catchment areas. LHAs plan and organize activities and interventions, and monitor the quality, appropriateness, and efficiency of the services provided [23]. Primary care is delivered by general practitioners (GPs), who operate under a national agreement as gatekeepers for drug prescriptions and access to specialty and hospital care. Based on this national agreement, the majority of GPs have been involved, since 2000, in the following monodisciplinary organizational arrangements: simple association, network, and group practice.

In Emilia-Romagna, a north-eastern region of Italy, primary care provided by LHAs has been organized, since 2007, into Primary Care Units (PCUs). Each PCU includes on average 16 GPs, according to their geographic proximity, as well as nurses, specialists, and other health professionals who provide multidisciplinary care. Specifically, GPs maintain their own individual or group practice and participate in meetings with the other PCU professionals to promote the sharing of knowledge and team work [24]. Since 2008, the LHA of Bologna has promoted in the PCUs the activation of a specific diagnostic/therapeutic pathway for the integrated management of patients with HF that involves GPs, nurses, and community or hospital cardiologists.

The aim of this paper was to evaluate the impact of different organizational factors, particularly at the primary care level, on unplanned readmissions of HF patients in the LHA of Bologna.

## Materials and Methods

### Setting and study population

In this observational retrospective cohort study, the population included residents of the LHA of Bologna (866,000 inhabitants in 2012) who were discharged from any Italian hospital with a primary diagnosis of HF (S1 Table for ICD-9-CM codes) from January 1, 2010 to December 31, 2010. Data were retrieved from the Hospital Discharge Records (HDRs) Database (in Italian, *scheda di dimissione ospedaliera* [SDO]). Repeated admissions within one day of discharge were regarded as a single “episode of care”, and the beginning of the follow-up period was set at the date of the hospital discharge for the index episode [25]. The length of individual follow-up was one year for all patients, except in the case of premature death (identified through the Regional Mortality Register Database).

Patients were excluded from the analysis if any of the following criteria were met:

1. Patients discharged with a primary or secondary diagnosis of HF in the previous two-year period (between January 1, 2008, and December 31, 2009), in order to try to include only incident cases of HF and to study patients being enrolled for the first time in the HF pathway during the study period;
2. A secondary diagnosis of acute renal failure (ICD-9-CM codes 584.5, 584.6, 584.7, 584.8, 584.9) or acute oedema of the lung, unspecified and not related to heart disease or failure (ICD-9-CM code 518.4), i.e., patients who have symptoms probably related to causes other than HF;
3. Age <60 years (5^th^ percentile), because younger patients may have different clinical features at diagnosis and survival, compared with older patients;
4. Length of stay for the episode of care >34 days (95^th^ percentile), i.e., very complex or unstable cases;
5. Patients whose GP stopped working before the end of the follow-up period (one year after discharge), in order to relate each of the patients to only one GP;
6. Patients who died during hospital stay, as they cannot experience rehospitalizations.

### Organizational factors

The organizational factors of interest included discharge from a cardiology department (vs. other departments), GP monodisciplinary organizational arrangement (simple association, network, or group practice), and implementation of a specific pathway of care for HF in the PCUs.

According to the national agreement, GP simple association consists in coordinating opening hours, implementing clinical-diagnostic guidelines for the most prevalent diseases, and holding regular meetings to review the quality of the activities and to promote the adoption of common prescriptive behaviours. In addition to the simple association features, GPs in a network share the electronic patient records and have access to the LHA system for the reservation of laboratory tests and specialty visits. Group practice implies, in addition to the network features, working in the same facility [26, 27].

In the HF care pathway promoted by the LHA of Bologna, skilled nurses participate in specific training sessions, GPs meet to share evidence-based guidelines, and fast tracks are activated for diagnostic tests when needed. Patients can be referred by GPs, or by hospital specialists when the diagnosis is made for the first time during hospitalization. When the disease is stable, the goal is to promote patient self-management through counselling by nurses to improve lifestyle and optimize therapy compliance, and the detection of early acute symptoms of HF. After discharge or HF worsening, patients are called periodically to assess their health status, and laboratory and instrumental tests monitoring visits with GPs and cardiologists are planned.

In addition, we examined the number of patients in the GP roster and, for each PCU, the number of GPs and the number of nurses’ weekly work hours. These data, including the presence or absence of a diagnostic/therapeutic pathway for HF, were retrieved from the Regional Primary Care Observatory Survey, which gathers information about the organizational processes and services of Emilia-Romagna. The number of patients in the GP roster, the number of GPs and the number of nurses’ weekly work hours were categorized, as provided in the Survey, into the following subgroups: <1000 vs. 1000–1499 vs. ≥1500 patients, ≤15 vs. >15 GPs, and ≤350 vs. >350 hours. The presence or absence of a diagnostic/therapeutic pathway for HF was cross-checked by interviewing the representatives of the PCUs of the LHA of Bologna.

### Outcome measure

The primary study outcome was the number of unplanned hospital readmissions for HF (S1 Table for ICD-9-CM codes). The secondary study outcome was the number of unplanned hospital readmissions for any reason. Both outcomes were measured in four different timeframes: 30 days (short-term), 90 days (medium-term), 180 days (mid-long-term), and 365 days (long-term). Information on hospital readmissions was obtained from the HDRs; repeated readmissions within one day of discharge were regarded as one single episode.

### Potential confounders

We considered a number of potential confounders measured before and/or during the index episode that may influence the risk of hospital readmission. Specifically, we considered patient gender and age, index episode length of stay, provision of intensive care, 22 comorbidities retrieved from HDRs for both the index episode and 2 years before (S2 Table for ICD-9-CM codes), and the prolonged use of specific drugs during the 12 months prior to the index admission (defined as at least 3 filled prescriptions on different dates, in line with [28]) (S3 Table for ATC codes and data source). We also considered as potential confounders some GP characteristics retrieved from the Regional Register of General Practitioners using December 31, 2010 as the reference date, and linked with patient HDRs using the GP identification code. Specifically, we investigated gender, age, and ambulatory location (i.e., urban, rural, or mountain area).

### Statistical analysis

We investigated the association between organizational determinants and hospital readmissions using multilevel Poisson regression analyses [29]. Given the hierarchical structure of our data, with patients nested into GPs and GPs nested into PCUs, we fitted three-level hierarchical regression models; the first level was patients, the second GPs, and the third PCUs. We opted for a multilevel analysis in order to account for the non-independence of observations within groups. The confounders to be included in the multilevel models were identified in a preliminary stepwise Poisson regression (level of removal = 5%, level of entry = 5%) to avoid over-parameterization and improve estimator efficiency [30]. The confounders included in the final models are reported in the table footnotes and presented in S4 and S5 Tables.

Statistical analyses were performed using Stata software, version 13 (StataCorp. 2013. *Stata Statistical Software*: *Release 13*. College Station, TX: StataCorp LP).

### Ethics statement

The study is exempt from approval from the Ethics Committee of the LHA of Bologna. It was conducted in conformity with the regulations for data management from the Regional Health Authority of Emilia-Romagna, and with the Italian *Code of conduct and professional practice applying to processing of personal data for statistical and scientific purposes* (art. 20–21, legislative decree 196/2003) (http://www.garanteprivacy.it/web/guest/home/docweb/-/docweb-display/docweb/1115480) published in the Official Journal no. 190 of August 14, 2004, which explicitly exempts the need for approval from the Ethics Committee when using anonymous data (Preamble #8).

In Italy, anonymous administrative data-gathering is subject to the law *Protection of individuals and other subjects with regard to the processing of personal data*, *ACT no*. *675 of 31*.*12*.*1996* (amended by Legislative Decree no. 123 of 09.05.1997, no. 255 of 28.07.1997, no. 135 of 08.05.1998, no. 171 of 13.05.1998, no. 389 of 6.11.1998, no. 51 of 26.02.1999, no. 135 of 11.05.1999, no. 281 of 30.07.1999, no. 282 of 30.07.1999 and no. 467 of 28.12.2001) (http://www.privacy.it/legge675encoord.html).

Data were anonymized prior to the analysis at the regional statistical office, and each patient was assigned a unique identifier that eliminates the ability to trace the patient’s identity or other sensitive data. As anonymized administrative data are used routinely for health care management, no specific written informed consent was needed to use the patient information.

## Results

### Study patients

**Fig 1:**
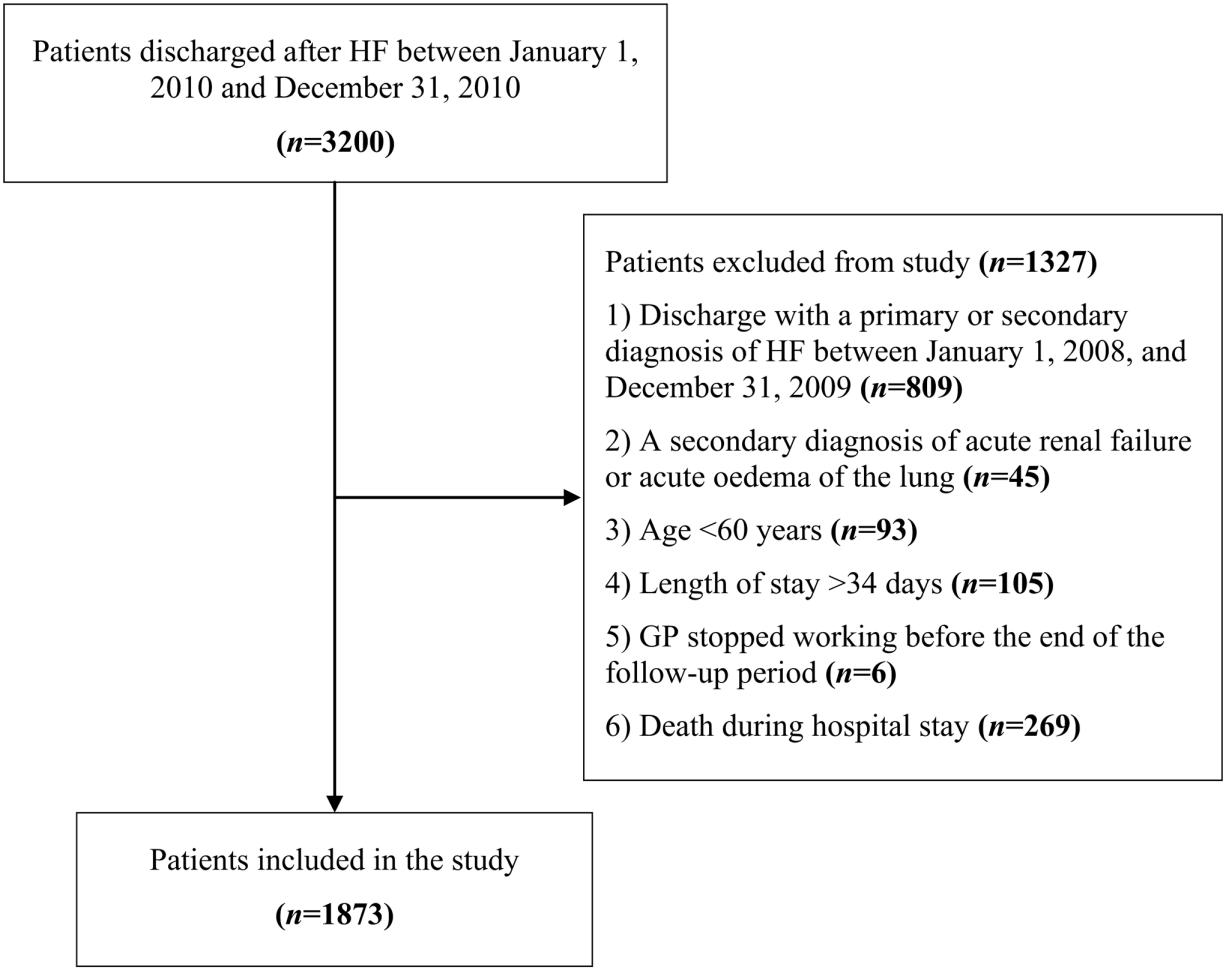
Patients’ flow diagram. *Abbreviations*: HF, heart failure.

Of the 3200 patients discharged after HF, 1873 (58.5%) met inclusion criteria (Fig 1). The median age was 83 years (interquartile range [IQR] = 77–88) and median hospital stay was 8 days (IQR = 5–12); 1040 (55.5%) were females and 564 (26.1%) died during the follow-up period, with an overall incidence rate of 0.9/1000 person-days (Table 1). Most patients (88.5%) were discharged from non-cardiology departments, 30.5% were over 86 years of age, 51 (2.7%) needed intensive care during hospitalization (of these, 41 were discharged from a cardiology department), and almost half (45.9%) had at least two comorbidities. Among the comorbidities retrieved from the HDRs, the most frequent was hypertension (25.6%), followed by other forms of ischemic heart disease (25.0%), chronic nephropathies (18.4%), and conduction disorders and cardiac dysrhythmias (18.2%). The majority of patients had used antihypertensive drugs before the acute HF episode (84.7%), and half of subjects had used antiplatelet drugs (44.3%). Of the 1873 study patients, 974 (52.0%) were readmitted to the hospital for any reason during a year of follow-up: 407 (21.7%) for non-cardiovascular causes, 376 (20.1%) for HF, and 191 (10.2%) for cardiovascular causes other than HF. The most frequent non-cardiovascular causes of readmission were pneumonia, bronchitis, chronic bronchitis, acute respiratory failure, and acute kidney failure. Of the 974 patients rehospitalized for any reason, 248 (25.5%) experienced an additional rehospitalization, 100 (10.3%) two additional rehospitalizations, and 82 (8.4%) three or more. The total number of hospital readmissions for HF and all causes are summarized in Table 2.

**Table 1:** Characteristics of the study patients.

	*N = 1873*	*%*
**Gender**		
Male	833	44.5
Female	1040	55.5
**Age (years)**		
<80	637	34.0
80–86	665	35.5
>86	571	30.5
**Length of hospital stay (days)**		
<7	720	38.4
7–10	549	29.3
>10	604	32.3
**Discharge from a cardiology department**		
No	1658	88.5
Yes	215	11.5
**Provision of intensive care**		
No	1822	97.3
Yes	51	2.7
**Number of comorbidities**		
0	531	28.4
1	482	25.7
≥2	860	45.9
**Specific comorbidities**		
Malignant tumours	173	9.2
Diabetes	196	10.5
Disorders of lipoid metabolism	77	4.1
Obesity	60	3.2
Hematologic diseases	129	6.9
Hypertensive diseases	480	25.6
Old AMI	158	8.4
Other forms of ischemic heart disease	469	25.0
Ill-defined descriptions and complications of heart disease	27	1.4
Rheumatic heart disease	76	4.1
Cardiomyopathies	116	6.2
Other cardiac diseases	106	5.7
Conduction disorders and cardiac dysrhythmias	340	18.2
Cerebrovascular diseases	248	13.2
Vascular diseases	154	8.2
COPD	204	10.9
Chronic nephropathies	345	18.4
Chronic diseases of liver, pancreas and intestine	47	2.5
Old bypass	43	2.3
Old PCI	59	3.2
Other surgery of the heart	27	1.4
Other surgery of great vessels	45	2.4
**Drug use 12 months before admission (≥3 prescriptions)**		
Antidiabetic drugs	356	19.0
Drugs for cardiac therapy	508	27.1
Drugs for obstructive airway diseases	273	14.6
Antihypertensive drugs	1587	84.7
Statins	441	23.6
Antiplatelet drugs	830	44.3

*Abbreviations*: AMI, acute myocardial infarction; COPD, chronic obstructive pulmonary disease; PCI, percutaneous coronary intervention.

**Table 2:** Counts and incidence rates of hospital readmissions during the follow-up period, overall and for heart failure.

Follow-up period	Readmissions for heart failure		Readmissions for any reason	
	N	IR	N	IR
		(×1000 person-days)		(×1000 person-days)
Short-term	88	1.6	300	5.5
Medium-term	190	1.2	633	4.0
Mid-long-term	313	1.0	1045	3.4
Long-term	498	0.9	1734	3.0

*Abbreviations*: IR, incidence rate.

### GPs and PCUs

A total of 541 GPs and 41 PCUs were observed (Table 3). Half of the GPs operated in group practice (51.0%), and about one third had a roster of more than 1500 patients (36.2%). Almost half of the GPs operated in rural areas, 45.7% in the city of Bologna, and 6.3% in the mountain areas. Of the 41 PCUs, 21 had implemented a specific diagnostic/therapeutic pathway for HF. The mean number of HF patients per GP was 3.9 ± 2.4, and the mean number of GPs per PCU was 13.5 ± 4.1.

**Table 3:** GP and PCU characteristics.

*GP characteristics*	*N = 541*	*%*
**Gender**		
Male	173	32.0
Female	368	68.0
**Age (years)**		
≤50	78	14.4
51–55	138	25.5
56–60	241	44.6
>60	84	15.5
**Organizational arrangement**		
None	134	24.8
Simple association	5	0.9
Network	126	23.3
Group practice	276	51.0
**Number of patients in the roster**		
<1000	109	20.2
1000–1499	236	43.6
≥1500	196	36.2
**Ambulatory location**		
Urban area	247	45.7
Rural area	260	48.1
Mountain area	34	6.3
***PCU characteristics***	***N = 41***	***%***
**Number of GPs**		
≤15	24	58.5
>15	17	41.5
**Number of nurses’ weekly working hours**		
≤350	25	61.0
>350	16	39.0
**Diagnostic/therapeutic pathway for heart failure patients**		
No	20	48.8
Yes	21	51.2

*Abbreviations*: GP, general practitioner; PCU, primary care unit.

### Impact of organizational factors on hospital readmissions

The results of regression models, showing factors in relation to hospital readmissions for HF and all causes, are presented in Tables 4 and 5, respectively. After adjusting for confounders (S4 and S5 Tables), the presence of a HF care pathway in the PCU was the only factor significantly associated with a lower risk of readmission for HF in the short-, medium-, mid-long- and long-term period (short-term: IRR [incidence rate ratio] = 0.57, 95% CI [confidence interval] = 0.35–0.92; medium-term: IRR = 0.70, 95% CI = 0.51–0.96; mid-long-term: IRR = 0.79, 95% CI = 0.64–0.98; long-term: IRR = 0.82, 95% CI = 0.67–0.99), and with a lower risk of all-cause readmission in the short-term period (IRR = 0.73, 95% CI = 0.57–0.94).

**Table 4:** Adjusted associations of organizational determinants with readmissions for heart failure, expressed in terms of incidence rate ratios (IRRs) derived from multilevel Poisson regression models.

Variables	Short-term			Medium-term			Mid-long-term			Long-term		
	IRR^a^	95% CI	*P* value	IRR^b^	95% CI	*P* value	IRR^c^	95% CI	*P* value	IRR^d^	95% CI	*P* value
***Patient characteristics***												
**Discharge from a cardiology department**												
No	1.00			1.00			1.00			1.00		
Yes	0.51	0.22–1.17	0.114	0.91	0.58–1.44	0.687	1.15	0.67–1.97	0.623	0.95	0.60–1.48	0.811
***GP characteristics***												
**Organizational arrangement**												
None/Simple association	1.00			1.00			1.00			1.00		
Network	1.09	0.63–1.91	0.751	1.22	0.82–1.80	0.324	1.10	0.76–1.59	0.599	1.10	0.82–1.47	0.539
Group practice	1.28	0.68–2.40	0.449	1.31	0.85–2.04	0.223	1.26	0.82–1.92	0.295	1.16	0.80–1.69	0.438
**Number of patients in the roster**												
<1000	1.00			1.00			1.00			1.00		
1000–1499	0.94	0.47–1.85	0.849	1.06	0.65–1.74	0.804	1.06	0.72–1.56	0.768	1.24	0.87–1.78	0.236
≥1500	0.90	0.45–1.79	0.757	1.09	0.66–1.78	0.738	0.85	0.54–1.36	0.501	0.99	0.67–1.47	0.952
***PCU characteristics***												
**Number of GPs**												
≤15	1.00			1.00			1.00			1.00		
>15	1.19	0.69–2.07	0.535	1.11	0.78–1.59	0.564	1.01	0.76–1.36	0.932	0.97	0.73–1.28	0.822
**Number of nurses’ weekly working hours**												
≤350	1.00			1.00			1.00			1.00		
>350	0.97	0.55–1.72	0.915	0.88	0.61–1.28	0.508	0.80	0.61–1.07	0.128	0.90	0.70–1.17	0.431
**Diagnostic/therapeutic pathway for heart failure**												
No	1.00			1.00			1.00			1.00		
Yes	0.57	0.35–0.92	0.020	0.70	0.51–0.96	0.026	0.79	0.64–0.98	0.029	0.82	0.67–0.99	0.043
**Pseudo *R*** ^***2***^ ^**e**^		5.5%			4.8%			7.2%			9.3%	

^a^Adjusted for significant covariates, including: other forms of ischemic heart disease, chronic nephropathies, and previous use of drugs for cardiac therapy.

^b^Adjusted for significant covariates, including: patients’ age, diabetes, cardiomyopathies, chronic nephropathies, and previous use of drugs for cardiac therapy.

^c^Adjusted for significant covariates, including: patients’ age, diabetes, COPD, chronic nephropathies, and previous use of drugs for cardiac therapy.

^d^Adjusted for significant covariates, including: patients’ age, diabetes, other cardiac diseases, COPD, chronic nephropathies, and previous use of antihypertensive drugs.

^e^Pseudo *R* ^*2*^ indicates how much of the variation of the phenomenon was explained by the covariates included in the model.

*Abbreviations*: IRR, incidence rate ratio; 95% CI, 95% confidence interval.

**Table 5:** Adjusted associations of organizational determinants with readmissions for any reason, expressed in terms of incidence rate ratios (IRRs) derived from multilevel Poisson regression models.

Variables	Short-term			Medium-term			Mid-long-term			Long-term		
	IRR^a^	95% CI	*P* value	IRR^b^	95% CI	*P* value	IRR^c^	95% CI	*P* value	IRR^d^	95% CI	*P* value
***Patient characteristics***												
**Discharge from a cardiology department**												
No	1.00			1.00			1.00			1.00		
Yes	0.70	0.46–1.07	0.099	0.91	0.70–1.20	0.511	1.02	0.75–1.38	0.918	0.87	0.68–1.10	0.228
***GP characteristics***												
**Organizational arrangement**												
None/Simple association	1.00			1.00			1.00			1.00		
Network	0.98	0.73–1.31	0.868	0.98	0.79–1.22	0.875	1.07	0.89–1.27	0.512	1.04	0.91–1.20	0.528
Group practice	0.98	0.70–1.38	0.924	1.00	0.78–1.28	0.979	1.14	0.89–1.46	0.297	1.12	0.93–1.37	0.239
**Number of patients in the roster**												
<1000	1.00			1.00			1.00			1.00		
1000–1499	1.05	0.73–1.52	0.792	0.97	0.75–1.27	0.843	1.05	0.81–1.37	0.719	1.18	0.91–1.53	0.222
≥1500	0.91	0.63–1.34	0.642	0.94	0.72–1.23	0.655	0.95	0.73–1.25	0.727	1.05	0.82–1.35	0.701
***PCU characteristics***												
**Number of GPs**												
≤15	1.00			1.00			1.00			1.00		
>15	1.08	0.81–1.44	0.607	1.00	0.81–1.23	0.999	0.98	0.81–1.18	0.812	0.96	0.84–1.09	0.516
**Number of nurses’ weekly working hours**												
≤350	1.00			1.00			1.00			1.00		
>350	0.92	0.68–1.24	0.577	1.00	0.81–1.24	0.986	0.98	0.80–1.19	0.827	1.02	0.87–1.19	0.842
**Diagnostic/therapeutic pathway for heart failure**												
No	1.00			1.00			1.00			1.00		
Yes	0.73	0.57–0.94	0.013	0.85	0.71–1.02	0.087	0.90	0.76–1.06	0.212	0.91	0.78–1.06	0.239
**Pseudo *R*** ^***2***^		3.7%			4.6%			6.3%			8.3%	

^a^Adjusted for significant covariates, including: patients’ age, length of hospital stay, malignant tumours, hypertensive diseases, chronic nephropathies, previous use of antidiabetic drugs, and previous use of drugs for cardiac therapy.

^b^Adjusted for significant covariates, including: patients’ age, length of hospital stay, malignant tumours, diabetes, cerebrovascular diseases, chronic nephropathies, chronic diseases of liver, pancreas and intestine, and previous use of drugs for cardiac therapy.

^c^Adjusted for significant covariates, including: patients’ age, length of hospital stay, malignant tumours, diabetes, cerebrovascular diseases, COPD, chronic nephropathies, chronic diseases of liver, pancreas and intestine, and previous use of drugs for cardiac therapy.

^d^Adjusted for significant covariates, including: patients’ age, length of hospital stay, malignant tumours, diabetes, old AMI, other cardiac diseases, cerebrovascular diseases, vascular diseases, COPD, chronic nephropathies, chronic diseases of liver, pancreas and intestine, and previous use of antihypertensive drugs.

Conversely, the presence of a HF care pathway was unrelated to the risk of all-cause readmission in the medium-, mid-long- and long-term period. No relationship was found between GP organizational arrangement and readmissions.

## Discussion

The main result of this observational study is that, after adjusting for age and significant comorbidities, the presence of a specific HF multidisciplinary care pathway was associated with a lower risk of hospital readmission for HF in the short- (30 days), medium- (90 days), mid-long- (180 days), and long-term (365 days) period. This pathway was also associated with a lower risk of all-cause readmission in the short-term period. Of note, we found a very high rate of all-cause 1-year readmissions (slightly above 50%), with just one third for another episode of HF; this finding is in line with those of Bottle [31] and Tuppin [32], and underscores the complexity of the health burden related to HF, typical of very old people with several comorbidities.

A number of studies have highlighted the effectiveness of multidisciplinary interventions on readmissions for HF and all causes [20,22,33,34]. The disease management of HF focuses on treatment monitoring using evidence-based recommendations to improve medication adherence and early identification of signs of HF worsening [35,36]. Specific interventions, similar to those promoted by the LHA of Bologna, are based on: a collaboration between community and hospital professionals [37], follow-up visits shortly after discharge [38,39], the presence of skilled nurses in the community, a patient and caregiver education about drug therapy, diet and the factors precipitating HF, and easy access to care [40]. These interventions can have, in addition, a positive psychological impact on patients [38].

The HF care pathway promoted by the LHA of Bologna is associated with a decreasing risk of readmission for both HF and all causes within 30 days of discharge. Achieving a reduction in 30-day readmissions is an important goal for health care systems: a number of hospital-based interventions, in fact, have been implemented to prevent rehospitalizations, but it has still not been determined which intervention or bundle of interventions is more effective [41,42]. Our results suggest that, in the management of patients with HF, multidisciplinary interventions involving not only hospital but also primary care professionals could improve quality of care in the early post-discharge period.

However, some readmissions are not preventable [43], and this could explain why the specific care pathway for HF promoted by the LHA of Bologna was not associated with a reduction of readmissions for all causes, at least in the medium- and long-term period. As indicated by the data presented in this study, our epidemiological context is characterized by an aging population suffering from multiple chronic conditions. Thus, the pathway focussing on a single disease may not respond effectively and efficiently to the needs of patients with multimorbidity [44].

Another finding of this study is that GP monoprofessional organizational arrangement was unrelated with hospital readmissions of HF patients. It is likely that, after adjusting for significant patient characteristics and the presence of specific HF care pathways in the PCUs, this organizational factor does not provide additional independent information on the risk of hospital readmission. Moreover, GP organizational arrangement may influence the good management of chronic conditions, such as diabetes, with the potential effect of reducing adverse outcomes (i.e., hospital admissions for diabetes complications) [26], but for a more complex condition, such as HF, monoprofessional practice is likely not enough.

The results of the present study should be interpreted in light of its strengths and limitations. Methodological strengths include the adjustment of the association between organizational factors and readmission for a variety of patient clinical and demographic characteristics (age, sex, comorbidities, etc.), and other characteristics recorded at the index hospitalization (length of stay and provision of intensive care) as proxies of disease severity. Limitations include the use of the HDR Database, which does not include variables such as patient lifestyle behaviours (smoking, diet, and physical activity), or other relevant clinical information. Moreover, the Primary Care Observatory of Emilia-Romagna considers only a limited number of variables which are already grouped into classes when the survey is filled out by the representatives of each PCU, and information on inclusion of individual patients in the HF pathway is not available. Also, we are aware that the high number of exclusion criteria may have limited the external validity of the study; however, when analyses were extended to younger patients, longer lengths of hospital stay and patients with a secondary diagnosis of acute renal failure or acute oedema of the lung, results did not change appreciably (data not presented). Lastly, our study was based on HF patients living in the LHA of Bologna, the capital of the Emilia-Romagna region. However, this study included all patients from one of the largest Italian LHAs, and there is no reason to think that our findings would not be generalizable to other regions or countries with a health care delivery system and population composition similar to those of Northern Italy.

In conclusion our study shows that the HF care specific pathway implemented at the primary care level was associated with lower readmission rate for HF in each timeframe, and also with lower readmission rate for all causes in the short-term period; this evidence suggests that the engagement of primary care professionals starting from the early post-discharge period may be relevant in the management of patients with HF. This pathway, however, was unrelated with all-cause readmissions in the medium- and long-term period, suggesting that multidisciplinary interventions focussing on a single disease may not respond to the needs of an aging population that suffers from multiple chronic conditions. Our findings may be useful to policymakers and health care managers for the development of good quality and sustainable care services for the elderly population.

Further research should focus on both readmission and the competing risk of post-discharge mortality, in an effort to investigate the overall impact of organizational factors on patient safety and quality of care. More studies are also warranted to identify the key elements of multidisciplinary approaches that are more effective for elderly patients with multimorbidity, and to evaluate the cost-effectiveness of these primary care organizational models.

## Supporting Information

S1 Table ICD-9-CM diagnosis codes for identification of heart failure incident cases and hospital readmissions. (PDF)

S2 Table ICD-9-CM codes for identification of comorbid conditions from hospital discharge records. (PDF)

S3 Table Medication use over 12 months before heart failure using Outpatient Pharmaceutical Database. (PDF)

S4 Table Confounding variables for heart failure hospital readmissions estimated by multilevel Poisson regression models. (PDF)

S5 Table Confounding variables for all-cause hospital readmissions estimated by multilevel Poisson regression models. (PDF)

S1 Dataset Supporting data. (XLS)

## References

[1] Mosterd A, Hoes AW. Clinical epidemiology of heart failure. Heart. 2007;93: 1137–1146. 17699180 10.1136/hrt.2003.025270 PMC1955040

[2] McMurray JJ, Adamopoulos S, Anker SD, Auricchio A, Böhm M, Dickstein K, et al ESC Guidelines for the diagnosis and treatment of acute and chronic heart failure 2012: The Task Force for the Diagnosis and Treatment of Acute and Chronic Heart Failure 2012 of the European Society of Cardiology. Developed in collaboration with the Heart Failure Association of the ESC (HFA). Eur J Heart Fail. 2012;14: 803–869. 10.1093/eurjhf/hfs105 22828712

[3] Wagner EH. Chronic disease management: what will it take to improve care for chronic illness? Eff Clin Pract. 1998;1: 2–4. 10345255

[4] Bodenheimer T, Wagner EH, Grunbach K. Improving primary care for patients with chronic illness. JAMA. 2002;288: 1775–1779. 12365965 10.1001/jama.288.14.1775

[5] Bodenheimer T, Wagner EH, Grunbach K. Improving primary care for patients with chronic illness: the chronic care model, Part 2. JAMA. 2002;288: 1909–1914. 12377092 10.1001/jama.288.15.1909

[6] Coleman K, Austin BT, Brach C, Wagner EH. Evidence on the Chronic Care Model in the new millennium. Health Aff (Millwood). 2009;28: 75–85. 10.1377/hlthaff.28.1.75 19124857 PMC5091929

[7] Ham C. The ten characteristics of the high-performing chronic care system. Health Econ Policy Law. 2010;5: 71–90. 10.1017/S1744133109990120 19732475

[8] Capomolla S, Febo O, Ceresa M, Caporotondi A, Guazzotti G, La Rovere M, et al Cost/utility ratio in chronic heart failure: comparison between heart failure management program delivered by day-hospital and usual care. J Am Coll Cardiol. 2002;40: 1259–1566. 12383573 10.1016/s0735-1097(02)02140-x

[9] Del Sindaco D, Pulignano G, Minardi G, Apostoli A, Guerrieri L, Rotoloni M, et al Two-year outcome of a prospective, controlled study of a disease management programme for elderly patients with heart failure. J Cardiovasc Med (Hagerstown). 2007;8: 324–329. 17443097 10.2459/JCM.0b013e32801164cb

[10] Ducharme A, Doyon O, White M, Rouleau JL, Brophy JM. Impact of care at a multidisciplinary congestive heart failure clinic: A randomized trial. CMAJ. 2005;173: 40–45. 15997043 10.1503/cmaj.1041137 PMC1167811

[11] Kasper E K, Gerstenblith G, Hefter G, Van Anden E, Brinker JA, Thiemann DR, et al A randomized trial of the efficacy of multidisciplinary care in heart failure outpatients at high risk of hospital re-admission. J Am Coll Cardiol. 2002;39: 471–480. 11823086 10.1016/s0735-1097(01)01761-2

[12] Tsuyuki RT, Fradette M, Johnson JA, Bungard TJ, Eurich DT, Ashton T, et al A multicenter disease management program for hospitalized patients with heart failure. J Card Fail. 2004;10: 473–480. 15599837 10.1016/j.cardfail.2004.02.005

[13] Blue L, Lang E, McMurray JJ, Davie AP, McDonagh TA, Murdoch DR, et al Randomised controlled trial of specialist nurse intervention in heart failure. BMJ. 2001;323: 715–718. 11576977 10.1136/bmj.323.7315.715 PMC56888

[14] Krumholz HM, Amatruda J, Smith G L, Mattera JA, Roumanis SA, Radford MJ, et al Randomized trial of an education and support intervention to prevent readmission of patients with heart failure. J Am Coll Cardiol. 2002;39:83–89. 11755291 10.1016/s0735-1097(01)01699-0

[15] Naylor MD, Brooten DA, Campbell RL, Maislin G, McCauley KM, Schwartz JS. Transitional Care of Older Adults Hospitalized with Heart Failure: A Randomized, Controlled Trial. J Am Geriatr Soc. 2004;52: 675–684. 15086645 10.1111/j.1532-5415.2004.52202.x

[16] Clark RA, Inglis SC, McAlister FA, Cleland JG, Stewart S. Telemonitoring or structured telephone support programmes for patients with chronic heart failure: systematic review and meta-analysis. BMJ. 2007; 334:942 17426062 10.1136/bmj.39156.536968.55 PMC1865411

[17] Klersy C, De Silvestri A, Gabutti G, Regoli F, Auricchio A. A meta-analysis of remote monitoring of heart failure patients. J Am Coll Cardiol. 2009;54: 1683–1694 10.1016/j.jacc.2009.08.017 19850208

[18] Gonseth J, Guallar-Castillón P, Banegas JR, Rodríguez-Artalejo F. The effectiveness of disease management programmes in reducing hospital re-admission in older patients with heart failure: a systematic review and meta-analysis of published reports. Eur Heart J. 2004;25: 1570–1595. 15351157 10.1016/j.ehj.2004.04.022

[19] Jaarsma T, Brons M, Kraai I, Luttik ML, Stromberg A. Components of heart failure management in home care; a literature review. Eur J Cardiovasc Nurs. 2013;12: 230–241. 10.1177/1474515112449539 22707520

[20] Takeda A, Taylor SJC, Taylor RS, Khan F, Krum H, Underwood M. Clinical service organisation for heart failure. Cochrane Database Syst Rev. 2012;9: CD002752 10.1002/14651858.CD002752.pub3 22972058

[21] Clark AM, Thompson DR. The future of the management programmes for heart failure. Lancet. 2008;372: 784–786. 10.1016/S0140-6736(08)61317-3 18774405

[22] Kul S, Barbieri A, Milan E, Montag I, Vanhaecht K, Panella M. Effects of care pathways on the in-hospital treatment of heart failure: a systematic review. BMC Cardiovasc Disord. 2012;12: 81 10.1186/1471-2261-12-81 23009030 PMC3507726

[23] Lo Scalzo A, Donatini A, Orzella L, Cicchetti A, Profili S, Maresso A. Italy: health system review. Health Syst Transit. 2009;11: 1–216.

[24] Armeni P, Compagni A, Longo F. Multiprofessional Primary Care Units: What Affects the Clinical Performance of Italian General Practitioners? Med Care Res Rev. 2014;71: 315–336. 24993251 10.1177/1077558714536618

[25] Kirchmayer U, Agabiti N, Belleudi V, Davoli M, Fusco D, Stafoggia M, et al Socio-demographic differences in adherence to evidence-based drug therapy after hospital discharge from acute myocardial infarction: a population-based cohort study in Rome, Italy. J Clin Pharm Ther. 2012;37: 37–44. 10.1111/j.1365-2710.2010.01242.x 21294760

[26] Fantini MP, Compagni A, Rucci P, Mimmi S, Longo F. General practitioners’ adherence to evidence-based guidelines: a multilevel analysis. Health Care Manage Rev. 2012;37: 67–76. 10.1097/HMR.0b013e31822241cf 21712723

[27] Visca M, Donatini A, Gini R, Federico B, Damiani G, Francesconi P, et al Group versus single handed primary care: a performance evaluation of the care delivered to chronic patients by Italian GPs. Health Policy. 2013;113: 188–198. 10.1016/j.healthpol.2013.05.016 23800605

[28] Lenzi J, Rucci P, Castaldini I, Protonotari A, Di Pasquale G, Di Martino M, et al Does age modify the relationship between adherence to secondary prevention medications and mortality after acute myocardial infarction? A nested case-control study. Eur J Clin Pharmacol. 2015;71: 243–250. 10.1007/s00228-014-1793-8 25529226

[29] Diez-Rioux AV. Multilevel analysis in public health research. Annu Rev Public Health. 2000;21: 171–192. 10884951 10.1146/annurev.publhealth.21.1.171

[30] Kirchmayer U, Di Martino M, Agabiti N, Bauleo L, Fusco D, Belleudi V, et al Effect of evidence-based drug therapy on long-term outcomes in patients discharged after myocardial infarction: a nested case—control study in Italy. Pharmacoepidemiol Drug Saf. 2013;22: 649–657. 23529919 10.1002/pds.3430 PMC3746119

[31] Bottle A, Aylin P, Bell D. Effect of the readmission primary diagnosis and time interval in heart failure patients: analysis of English administrative data. Eur J Heart Fail. 2014;16: 846–853. 10.1002/ejhf.129 25044392

[32] Tuppin P, Cuerq A, de Peretti C, Fagot-Campagna A, Danchin N, Juillière Y, et al Two-year outcome of patients after a first hospitalization for heart failure: A national observational study. Arch Cardiovasc Dis. 2014;107: 158–168. 10.1016/j.acvd.2014.01.012 24662470

[33] Rich MW, Beckham V, Wittenberg C, Leven CL, Freedland KE, Carney RM. A multidisciplinary intervention to prevent the readmission of elderly patients with congestive heart failure. N Engl J Med. 1995;333: 1190–1195. 7565975 10.1056/NEJM199511023331806

[34] Panella M, Marchisio S, Demarchi ML, Manzoli L, Di Stanislao F. Reduced in-hospital mortality for heart failure with clinical pathways: the results of a cluster randomised controlled trial. Qual Saf Health Care. 2009;18: 369–373. 10.1136/qshc.2008.026559 19812099

[35] Garin N, Carballo S, Gerstel E, Lerch R, Meyer P, Zare M, et al Inclusion into a heart failure critical pathway reduces the risk of death or readmission after hospital discharge. Eur J Intern Med. 2012;23: 760–764. 10.1016/j.ejim.2012.06.006 23122393

[36] Michalsen A, König G, Thimme W. Preventable causative factors leading to hospital admission with decompensated heart failure. Heart. 1998;80: 437–441. 9930040 10.1136/hrt.80.5.437 PMC1728853

[37] Bradley EH, Curry L, Horwitz LI, Sipsma H, Wang Y, Walsh MN, et al Hospital strategies associated with 30-day readmission rates for patients with heart failure. Circ Cardiovasc Qual Outcomes. 2013;6: 444–450. 10.1161/CIRCOUTCOMES.111.000101 23861483 PMC3802532

[38] Inglis SC, Clark RA, McAlister FA, Stewart S, Cleland JG. Which components of heart failure programmes are effective? A systematic review and meta-analysis of the outcomes of structured telephone support or telemonitoring as the primary component of chronic heart failure management in 8323 patients: Abridged Cochrane Review. Eur J Heart Fail. 2011;13: 1028–1040. 10.1093/eurjhf/hfr039 21733889

[39] McAlister FA, Youngson E, Bakal JA, Kaul P, Ezekowitz J, van Walraven C. Impact of physician continuity on death or urgent readmission after discharge among patients with heart failure. CMAJ. 2013;185: E681–E689. 10.1503/cmaj.130048 23959284 PMC3787192

[40] McAlister FA, Stewart S, Ferrua S, McMurray JJ. Multidisciplinary strategies for the management of heart failure patients at high risk for admission: a systematic review of randomized trials. J Am Coll Cardiol. 2004;44: 810–819. 15312864 10.1016/j.jacc.2004.05.055

[41] Hansen LO, Young RS, Hinami K, Leung A, Williams MV. Interventions to reduce 30-day rehospitalisation: a systematic review. Ann Intern Med. 2011;155: 520–528. 10.7326/0003-4819-155-8-201110180-00008 22007045

[42] Linden A, Butterworth SW. A comprehensive hospital-based intervention to reduce readmissions for chronically ill patients: a randomized controlled trial. Am J Manag Care. 2014;20: 783–792. 25365681

[43] Weinberger M, Oddone EZ, Henderson WG. Does increased access to primary care reduce hospital readmissions? Veterans Affairs Cooperative Study Group on Primary Care and Hospital Readmission. N Engl J Med. 1996;334: 1441–1447. 8618584 10.1056/NEJM199605303342206

[44] Barnett K, Mercer SW, Norbury M, Watt G, Wyke S, Guthrie B. Epidemiology of multimorbidity and implications for health care, research, and medical education: a cross-sectional study. Lancet. 2012;380: 37–43. 10.1016/S0140-6736(12)60240-2 22579043

