# Supplementary material for: Hospital Readmissions of Patients with Heart Failure: The Impact of Hospital and Primary Care Organizational Factors in Northern Italy
Source: PLoS One. 2015 May 26;10(5):e0127796. doi: 10.1371/journal.pone.0127796 (PMC4444393; doi:10.1371/journal.pone.0127796)
Supplement: S1 Table — (PDF) [file pone.0127796.s001.pdf]

S1 Table. ICD-9-CM diagnosis codes for identification of heart failure incident cases and hospital readmissions.

ICD-9-CM code	Condition
402.01	Malignant hypertensive heart disease with heart failure
402.11	Benign hypertensive heart disease with heart failure
402.91	Unspecified hypertensive heart disease with heart failure
404.01	Hypertensive heart and chronic kidney disease, malignant, with heart failure and with chronic kidney disease stage I through stage IV, or unspecified
404.03	Hypertensive heart and chronic kidney disease, malignant, with heart failure and with chronic kidney disease stage V or end stage renal disease
404.11	Hypertensive heart and chronic kidney disease, benign, with heart failure and with chronic kidney disease stage I through stage IV, or unspecified
404.13	Hypertensive heart and chronic kidney disease, benign, with heart failure and chronic kidney disease stage V or end stage renal disease
404.91	Hypertensive heart and chronic kidney disease, unspecified, with heart failure and with chronic kidney disease stage I through stage IV, or unspecified
404.93	Hypertensive heart and chronic kidney disease, unspecified, with heart failure and chronic kidney disease stage V or end stage renal disease
428.0	Congestive heart failure, unspecified
428.1	Left heart failure
428.20	Systolic heart failure, unspecified
428.21	Acute systolic heart failure
428.22	Chronic systolic heart failure
428.23	Acute on chronic systolic heart failure
428.30	Diastolic heart failure, unspecified
428.31	Acute diastolic heart failure
428.32	Chronic diastolic heart failure
428.33	Acute on chronic diastolic heart failure
428.40	Combined systolic and diastolic heart failure, unspecified
428.41	Acute combined systolic and diastolic heart failure
428.42	Chronic combined systolic and diastolic heart failure
428.43	Acute on chronic combined systolic and diastolic heart failure
428.9	Heart failure, unspecified
