# Supplementary material for: Hospital Readmissions of Patients with Heart Failure: The Impact of Hospital and Primary Care Organizational Factors in Northern Italy
Source: PLoS One. 2015 May 26;10(5):e0127796. doi: 10.1371/journal.pone.0127796 (PMC4444393; doi:10.1371/journal.pone.0127796)
Supplement: S2 Table — (PDF) [file pone.0127796.s002.pdf]

S2 Table. ICD-9-CM codes for identification of comorbid conditions from hospital discharge records.

Condition	ICD-9-CM diagnosis codes	
	Heart failure episode	Hospital discharges in the previous two years
Malignant tumours	140.0–208.9, V10	140.0–208.9, V10
Diabetes		250.0–250.9
Disorders of lipoid metabolism		272
Obesity	278.0	278.0
Hematologic diseases	280–285, 288, 289	280–285, 288, 289
Hypertensive diseases		401–405
Old AMI	412	410, 412
Other forms of ischemic heart disease		411, 413, 414
Ill-defined descriptions and complications of heart disease		429
Rheumatic heart disease	393–398	391, 393–398
Cardiomyopathies	425	425
Other cardiac diseases	745, V15.1, V42.2, V43.2, V43.3, V45.0	745, V15.1, V42.2, V43.2, V43.3, V45.0
Conduction disorders and cardiac dysrhythmias		426, 427
Cerebrovascular diseases	433, 437, 438	430–432, 433, 434, 436, 437, 438
Vascular diseases	440–448 (except 441.1, 441.3, 441.5, 441.6, 444), 557.1	440–448, 557
COPD		491–492, 494, 496
Chronic nephropathies	582–583, 585–588	582–583, 585–588
Chronic diseases of liver, pancreas and intestine	571–572, 577.1–577.9, 555, 556	571–572, 577.1–577.9, 555, 556
Old bypass	V45.81	V45.81, 36.1
Old PCI	V45.82	V45.82, 36.0, 00.66
Other surgery of the heart		35, 37.0, 37.1, 37.3, 37.4, 37.5, 37.6, 37.9
Other surgery of great vessels		38–39.5 (except 38.01, 38.02, 38.5, 38.11, 38.12, 38.31, 38.32, 38.93)

Abbreviations: AMI, acute myocardial infarction; COPD, chronic obstructive pulmonary disease; PCI, percutaneous coronary intervention.
