# Supplementary material for: Hospital Readmissions of Patients with Heart Failure: The Impact of Hospital and Primary Care Organizational Factors in Northern Italy
Source: PLoS One. 2015 May 26;10(5):e0127796. doi: 10.1371/journal.pone.0127796 (PMC4444393; doi:10.1371/journal.pone.0127796)
Supplement: S3 Table — (PDF) [file pone.0127796.s003.pdf]

S3 Table. Medication use over 12 months before heart failure using Outpatient Pharmaceutical Database.

Medication	ATC codes
Antidiabetic drugs	A10
Drugs for cardiac therapy	C01
Drugs for obstructive airway diseases	R03
Antihypertensive drugs	C02 (antihypertensive drugs), C03 (diuretics), C07 (β-blockers), C08 (calcium channel blockers), C09 (ACEIs/ARBs)
Statins	C10AA
Antiplatelet drugs	B01AC

Abbreviations: ATC, Anatomical Therapeutic Chemical Classification System; ACEIs/ARBs, angiotensin-converting enzyme inhibitors/angiotensin receptor blockers.
