# Supplementary material for: Hospital Readmissions of Patients with Heart Failure: The Impact of Hospital and Primary Care Organizational Factors in Northern Italy
Source: PLoS One. 2015 May 26;10(5):e0127796. doi: 10.1371/journal.pone.0127796 (PMC4444393; doi:10.1371/journal.pone.0127796)
Supplement: S4 Table — (PDF) [file pone.0127796.s004.pdf]

S4 Table. Confounding variables for heart failure hospital readmissions estimated by multilevel Poisson regression models.

Variables	Short-term			Medium-term			Mid-long-term			Long-term		
	IRR	95% CI	*P* value	IRR	95% CI	*P* value	IRR	95% CI	*P* value	IRR	95% CI	*P* value
**Age (years)**												
<80				1.00			1.00			1.00		
80–86	–			1.50	1.04–2.16	0.028	1.47	1.11–1.95	0.007	1.35	1.08–1.68	0.008
>86	–			1.56	1.06–2.30	0.023	1.54	1.10–2.15	0.012	1.59	1.20–2.10	0.001
**Comorbidities**												
Diabetes	–			1.63	1.11–2.39	0.013	1.66	1.20–2.31	0.002	1.84	1.41–2.41	<0.001
Other forms of ischemic heart disease	1.56	1.01–2.43	0.047	–			–			–		
Cardiomyopathies	–			1.88	1.16–3.05	0.010	–			–		
Other cardiac diseases	–			–			–			1.81	1.24–2.64	0.002
Chronic obstructive pulmonary disease	–			–			1.67	1.19–2.35	0.003	1.64	1.24–2.17	0.001
Chronic nephropathies	2.01	1.28–3.15	0.003	2.26	1.67–3.06	<0.001	2.20	1.65–2.92	<0.001	1.87	1.45–2.40	<0.001
**Drug use 12 months before admission (≥3 prescriptions)**												
Drugs for cardiac therapy	2.09	1.36–3.20	0.001	1.54	1.15–2.07	0.004	1.45	1.22–1.72	<0.001	–		
Antihypertensive drugs	–			–			–			1.71	1.16–2.53	0.007

Abbreviations: IRR, incidence rate ratio; 95% CI, 95% confidence interval.
