# Supplementary material for: Hospital Readmissions of Patients with Heart Failure: The Impact of Hospital and Primary Care Organizational Factors in Northern Italy
Source: PLoS One. 2015 May 26;10(5):e0127796. doi: 10.1371/journal.pone.0127796 (PMC4444393; doi:10.1371/journal.pone.0127796)
Supplement: S5 Table — (PDF) [file pone.0127796.s005.pdf]

S5 Table. Confounding variables for all-cause hospital readmissions estimated by multilevel Poisson regression models.

Variables	Short-term			Medium-term			Mid-long-term			Long-term		
	IRR	95% CI	*P* value	IRR	95% CI	*P* value	IRR	95% CI	*P* value	IRR	95% CI	*P* value
**Age (years)**												
<80	1.00			1.00			1.00			1.00		
80–86	1.24	0.93–1.66	0.149	1.26	1.03–1.55	0.025	1.18	0.98–1.43	0.085	1.17	1.03–1.34	0.017
>86	1.51	1.12–2.04	0.008	1.58	1.28–1.95	<0.001	1.48	1.22–1.80	<0.001	1.43	1.20–1.70	<0.001
**Length of hospital stay (days)**												
<7	1.00			1.00			1.00			1.00		
7–10	1.24	0.93–1.66	0.142	1.18	0.97–1.45	0.100	1.09	0.93–1.28	0.290	1.15	1.01–1.30	0.038
>10	1.52	1.16–2.01	0.003	1.48	1.22–1.79	<0.001	1.32	1.11–1.58	0.002	1.28	1.09–1.50	0.002
**Comorbidities**												
Malignant tumours	1.68	1.21–2.34	0.002	1.67	1.32–2.11	<0.001	1.37	1.10–1.72	0.006	1.35	1.11–1.63	0.002
Diabetes	–			1.35	1.06–1.70	0.013	1.55	1.28–1.87	<0.001	1.52	1.25–1.85	<0.001
Hypertensive diseases	1.30	1.02–1.66	0.037	–			–			–		
Old AMI	–			–			–			1.27	1.04–1.55	0.022
Other cardiac diseases	–			–			–			1.45	1.10–1.91	0.009
Cerebrovascular diseases	–			1.29	1.04–1.60	0.021	1.41	1.15–1.73	0.001	1.39	1.18–1.65	<0.001
Vascular diseases	–			–			–			1.29	1.01–1.66	0.045
COPD	–			–			1.36	1.11–1.67	0.004	1.46	1.21–1.76	<0.001
Chronic nephropathies	1.50	1.16–1.95	0.002	1.34	1.11–1.63	0.002	1.34	1.16–1.55	<0.001	1.23	1.06–1.44	0.008
Chronic diseases of liver, pancreas and intestine	–			1.54	1.01–2.36	0.044	1.91	1.26–2.88	0.002	1.42	1.00–2.01	0.048
**Drug use 12 months before admission (≥3 prescriptions)**												
Antidiabetic drugs	1.38	1.05–1.81	0.022	–			–			–		
Drugs for cardiac therapy	1.37	1.08–1.74	0.010	1.31	1.10–1.55	0.002	1.23	1.08–1.39	0.001	–		
Antihypertensive drugs	–			–			–			1.32	1.09–1.59	0.004
